# A novel evolutionary method for parameter-free MEMS structural design and its application in piezoresistive pressure sensors

**DOI:** 10.1038/s41378-023-00596-y

**Published:** 2023-10-25

**Authors:** Qinggang Meng, Junbo Wang, Deyong Chen, Jian Chen, Bo Xie, Yulan Lu

**Affiliations:** 1 State Key Laboratory of Transducer Technology, Aerospace Information Research Institute, Chinese Academy of Sciences, 100190 Beijing, China; 2 School of Electronic, Electrical and Communication Engineering, University of Chinese Academy of Sciences, 100049 Beijing, China

**Keywords:** Electrical and electronic engineering, Sensors

## Abstract

In this paper, a novel simulation-based evolutionary method is presented for designing parameter-free MEMS structures with maximum degrees of freedom. This novel design method enabled semiautomatic structure evolution by weighing the attributes of each segment of the structure and yielded an optimal design after multiple iterations. The proposed method was utilized to optimize the pressure-sensitive diaphragm of a piezoresistive pressure sensor (PPS). Finite element method (FEM) simulations revealed that, in comparison to conventional diaphragms without islands and with square islands, the optimized diaphragm increased the stress by 10% and 16% and reduced the nonlinearity by 57% and 77%, respectively. These improvements demonstrate the value of this method. Characterization of the fabricated PPS revealed a high sensitivity of 8.8 mV V^−1^ MPa^−1^ and a low nonlinearity of 0.058% FS at 20 °C, indicating excellent sensor performance.

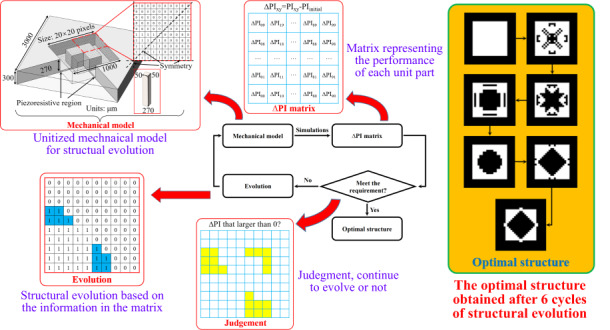

## Introduction

Microelectromechanical system (MEMS) devices, which are characterized by miniaturization, low cost, and mass production, play an important role in modern society due to their use in numerous fields, including aerospace, medical equipment, and industrial control^1–5^. Over time, industries have imposed more stringent performance requirements on MEMS devices. Thus, a primary objective of MEMS research has been the development of devices with better performance.

The physical characteristics and comprehensive performance of MEMS devices can be significantly influenced by their geometric structures. Conventional structural design requires a material design approach to incorporate a high degree of previous knowledge to obtain a set of control parameters for yielding the desired structure, such as the position, number, length, width and other parameters of microcolumns as used in microfluidic channel design^6^. Afterward, FEM simulation was utilized to individually optimize each of those parameters until a set of so-called optimal values that satisfied the objective were determined^7,8^. However, these artificial, parametric design approaches were highly reliant on designers and frequently had limited design flexibility and constrained geometries, which severely limited the performance of MEMS devices. In addition, for some complicated and multiparameter-controlled MEMS devices, it is difficult or perhaps impossible to simulate the structural parameters individually to obtain the optimum design.

To eliminate reliance on empirical knowledge and achieve the global optimum design, some efforts have attempted to implement various algorithms that aid in the design of MEMS devices. For example, Andojo Ongkodjojo et al. used simulated annealing (SA) to design a comb-drive single mass microgyroscope, achieved multiparameter automated optimization and significantly increased the sensitivity of the device^9^. However, SA can only optimize the parameters defined by formulas and thus can only determine a locally optimal solution costrainted by the limits of the formulas. Chen Wang et al. designed the free structure of a MEMS accelerometer and microgripper using a genetic algorithm (GA)^10,11^. After multiple generations of structural evolution, the performance of the devices was finally exceptional. Nonetheless, the structure developed by this method was still bound by parameters, limiting the design flexibility and device performance. In addition, the application of deep learning (DL) to develop MEMS resonators was also widespread. Other researchers used the structure of MEMS resonators and the properties derived through FEM simulation as training data to train the neural network, enabling the quick prediction of resonator performances and encouraging the design of MEMS structures without the need for prior knowledge^12–14^. Moreover, since the neural network is essentially a fast calculator, it cannot directly contribute to the development of new geometries or discover optimum designs.

To address these issues, based on FEM simulations, we propose a novel semiautomatic evolutionary method to achieve optimal MEMS structures. We describe a novel piezoresistive pressure sensor (PPS) with an optimal pressure-sensitive diaphragm that was designed and optimized by an evolutionary method as an example. Detailed modeling and optimization processes were conducted. Because the structure of the diaphragm was optimized with maximum degrees of freedom and no parameter limitations, the designed diaphragm exhibits sufficiently large stress and small deflection in comparison to other designs, thereby significantly enhancing the overall performance of the PPS. The method developed in this study is proposed to be an enabling tool in MEMS structure design.

## Optimization of the PPS island-diaphragm model by the evolutionary method

### Optimization goal

PPS is a type of sensor that utilizes the piezoresistive property of silicon to measure external pressure by producing an output voltage proportional to the input pressure^15^. The two most important characteristics of PPS are its sensitivity and output voltage nonlinearity, which determine its anti-interference performance and pressure measurement accuracy. Of all the sensor configurations, the pressure-sensitive diaphragm, which converts external pressure into measurable stress through its deflection, has the greatest impact on these two properties, where the greater the stress on the surface of the diaphragm under constant pressure (with all other parameters are held constant), the better the sensitivity of the sensor. Likewise, a smaller diaphragm deflection is accompanied by a reduced sensor nonlinearity^16^. Therefore, the design of the pressure-sensitive diaphragm is important for PPS.

The simplest diaphragm design has a flat structure, but presents surface stress and maximum deflection that are greatly constrained by each other. The improvement of surface stress can only be achieved by reducing the thickness of the diaphragm, which will increase its deflection significantly^17^. To overcome this tradeoff, a special diaphragm with a back-island structure was developed. A back-island with a particular shape can reduce the deflection while simultaneously concentrating and increasing the stress on the surface of the diaphragm^18^. Therefore, developing a structure with the smallest deflection and the largest surface stress, i.e., finding an ideal back-island structure that maximizes the stress under the same deflection, is the goal of diaphragm design.

To facilitate comparison and iterative optimization, the performance of the diaphragm must be quantified to a specific value. Assuming that the maximum surface stress on an island-diaphragm $x$ is ${\sigma }_{x}$ and corresponds to the average transverse-to-longitudinal stress difference in the piezoresistive region, the maximum deflection is ${\omega }_{x}$, and the stress-deflection relationship of a flat diaphragm without an island is $\sigma ={f}_{{\rm{flat}}}(\omega )$. Therefore, using the flat diaphragm as a comparison standard, the performance index (PI) of an island-diaphragm $x$ is produced as given below:

1 $${\rm{P}}{{\rm{I}}}_{x}=\frac{{\sigma }_{x}-{f}_{{\rm{flat}}}({\omega }_{x})}{{f}_{{\rm{flat}}}({\omega }_{x})}\times 100 \%$$

This formula demonstrates that the performance of an island-diaphragm is defined by the increase in stress relative to a flat diaphragm with the same deflection. Then, optimization can be performed with the aim of maximizing PI.

### Optimization method

**Fig. 1:**
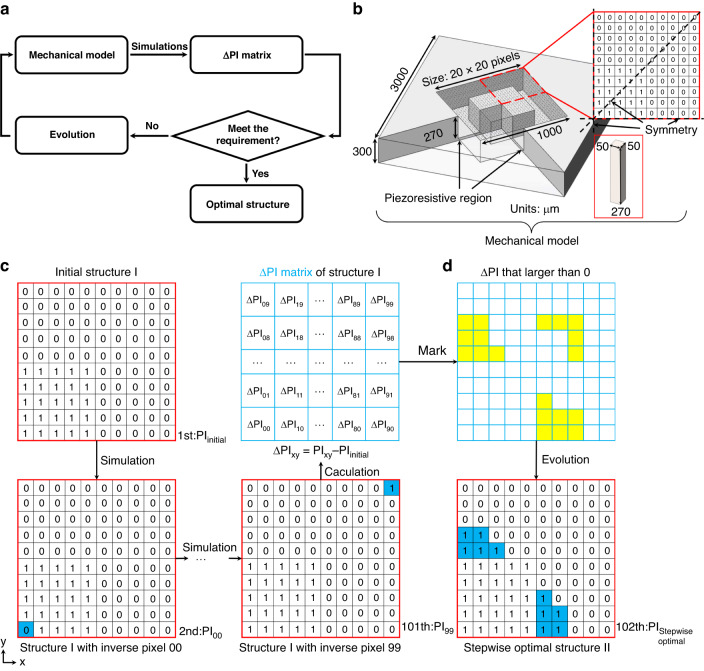
Optimization method. **a** Generic process flow for optimizing MEMS structures using the evolutionary method. **b** Schematic of the mechanical model of the PPS with island-diaphragm, the diaphragm region was depicted as a 20×20 pixel matrix in which 0 represents an empty element and 1 represents a solid element. The solid element was represents with dimensions of 50×50×270 μm in the inset. **c** Calculation of the ∆PI matrix which represents the influence of each pixel on the overall performance of diaphragm. **d** Structural evolution of the island-diaphragm based on the pixels larger than 0 in ∆PI matrix

As illustrated in Fig. 1a, a generic process flow for optimizing MEMS structures using the evolutionary method consists of three steps: the establishment of a mechanical model, the calculation of the ∆PI matrix, and structural evolution based on the ∆PI matrix. There is also a judgment in the middle step. If the ∆PI matrix meets the requirement, then the optimization process terminates. Otherwise, it continues to evolve, and the mechanical model is modified for a subsequent iteration.

Figure 1b shows a schematic of the mechanical model of the PPS with an island diaphragm, where the chip has dimensions of 3000 × 3000 × 300 μm and the pressure-sensitive diaphragm is separated into a flat portion with dimensions of 1000 μm × 1000 μm × 30 μm and an island portion with a height of 270 μm and an indeterminate structure. The piezoresistive region was located in the center of the edge on the back of the diaphragm. As the region to be optimized, the back space of the diaphragm is depicted as a 20 × 20 pixel matrix in which 0 represents an empty element and 1 represents a solid element. This matrix may represent up to 255 types of island-diaphragm structures (due to the characteristics of PPS, the matrix was distributed symmetrically along the four axes), and our goal was to determine the optimal structure from such a large number of configurations by the evolutionary method.

The subsequent optimization process was carried out through FEM simulation using COMSOL Multiphysics 5.6. Only a quarter of the full model was built in simulation for purposes of symmetry and simplification. Silicon with Young’s modulus of 170 GPa and a Poisson’s ratio of 0.28 was used as the material. The mechanical properties of the model were simulated using the “solid mechanics (solid)” interface, with a “fixed constraint” condition applied on the frame outside the diaphragm and a “boundary load” of 2 MPa applied to the surface of the diaphragm. The flat portion of the diaphragm was meshed with the “free triangular” node, while the island portion of the diaphragm was meshed with the “swept” node. Then, the stress-deflection relationship of the flat diaphragm (matrix = 0) was calculated by simulating and fitting the surface stress and deflection, as shown in Eq. 2, where the units of $\sigma$ and $\omega$ were MPa and μm, respectively. By substituting Eq. (2) into Eq. (1), the PI value of any island-diaphragm system can be determined:

2 $$\sigma \,={f}_{{\rm{flat}}}(\omega )=-132.8\cdot \omega \,{}^{0.6886}+14.61$$

The optimization procedure begins with a randomly chosen matrix with a performance index calculated as PI_initial_, as shown in Fig. 1c. Then, we invert the value of the 00th pixel (marked in blue in the figure) and run the second simulation to obtain a new performance index PI_00_ while preserving the values of the other pixels. For the island-diaphragm structure, the inversion of the pixel value means that if a solid element existed at the given location, it would be removed, and if none existed, it would be added. The deviation from the initial performance index $\varDelta {\rm{P}}{{\rm{I}}}_{00}={\rm{P}}{{\rm{I}}}_{00}-{\rm{P}}{{\rm{I}}}_{{\rm{initial}}}$ indicates how the 00th pixel affects the overall performance of the island-diaphragm system: if ΔPI_00_ is larger than 0, the change in this pixel is beneficial to the performance of the diaphragm, and the larger the value is, the greater the need for the change, and vice versa. ΔPI_00_ was then added to the 00th of the ΔPI matrix. Finally, we restore the 00th inverted pixel and invert the 01st pixel to obtain the second deviation value ΔPI_01_. Upon repeating this procedure 100 times, the ΔPI matrix was eventually filled.

The resulting ΔPI matrix shown in Fig. 1c contains much useful information about the present island-diaphragm structure. Notably, if all the values in this matrix were less than 0, the result would indicate that changing any section of the current structure would result in a decrease in performance, indicating that the existing structure was optimum, which was the judgment condition in Fig. 1a. Moreover, for a general matrix, the sign and magnitude of its value may lead to a deeper understanding of the present structure by revealing which parts of the structure were vital and effective (ΔPI_*xy*_⪡ 0), which were dispensable and inessential ($\varDelta {\rm{P}}{{\rm{I}}}_{xy}\approx 0$), and which were superfluous or destructive ($\varDelta {\rm{P}}{{\rm{I}}}_{xy}\gg 0$). Then, the structural evolution may be conducted under the guidance of the ΔPI matrix.

Figure 1d shows the structural evolution of the island-diaphragm system based on the pixels larger than 0 in the ΔPI matrix (as marked in yellow). The marks in the figure were introduced for illustrative purposes only and do not represent actual results. We note that the ΔPI matrix was derived by inverting each pixel separately and did not account for the interaction between pixels. Hence, if all pixels larger than 0 were inverted simultaneously, the unpredictable interaction would take precedence, and the performance of the diaphragm may not improve as anticipated or may even deteriorate. Thus, the ΔPI matrix can be used as a guide to the approximate optimization direction. When the degree of structural change rises, the guiding function of the original matrix is weakened. An effective solution was to invert only one pixel with the largest ΔPI_*xy*_ (which is actually two pixels, due to the symmetry) after acquiring a ΔPI matrix to avoid the effect of the interaction. Nevertheless, this iterative approach was too slow, resulting in an excessively lengthy simulation time, with an optimization step size that was so tiny that it was easy for the structure to reach an unsolvable local maximum. The ensuing evolution strategy was to follow the guidance of the ΔPI matrix and invert the pixels larger than 0 in order of ΔPI_*xy*_ from large to small until a structure with the largest PI was found. For example, after obtaining a ΔPI matrix, inverting four pixels with the largest ΔPI_*xy*_ produced a slightly changed diaphragm with a PI of 100, then inverting two of the remaining largest pixels resulted in a further changed diaphragm with a PI of 130, and inverting two more pixels yielded a PI of 120. Hence, the optimal structure was the second case, which indicated that the inverted pixels in the optimal structure were some but not all pixels with $\Delta {\rm{P}}{{\rm{I}}}_{xy}$ larger than 0, as shown in Fig. 1d. This strategy not only ensured the efficiency of the simulation but also prevented the local optimum to a significant degree. The final structure in Fig. 1d was the result of the stepwise optimal solution that maximized the use of the original ΔPI matrix. For further optimization, the mechanical model was modified according to with the stepwise optimal solution, and new simulations were conducted according to Fig. 1a.

### Optimization process

**Fig. 2:**
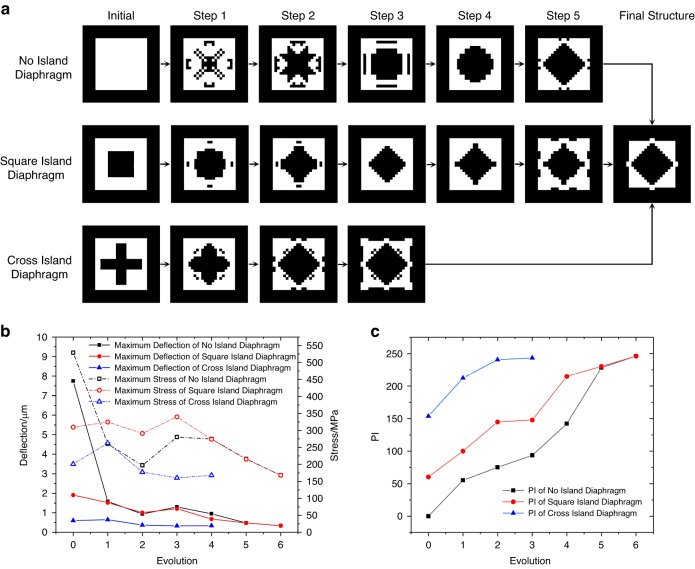
Optimization process. **a** Graphical representation of island-diaphragm structural changes for three different initial structures. **b** Maximum stress and deformation of three island-diaphragm structures during evolution. **c** PI of three island-diaphragm structures during evolution

Three different initial structures of the island-diaphragm were chosen for optimization using the evolution method, and a graphical representation of the changes is shown in Fig. 2a. The optimization procedure was performed six times for diaphragms with no islands and square islands and four times for cross-island diaphragms. Various initial structures resulted in identical optimization outcomes, demonstrating that the evolutionary method was insensitive to the initial condition and had a high probability of reaching the global optimum solution. Figure 2b shows the maximum stress and deflection of three island-diaphragm structures during evolution. As the number of evolutions increases, the maximum deflection of the island-diaphragm continues to decrease, indicating that the evolutionary method has functioned to improve the rigidity of the diaphragm. However, the stress on the island-diaphragm exhibited a general decreasing tendency that was accompanied by an increase at some points, revealing that the evolutionary method also functioned to raise the stress (although this was not a dominating outcome). Figure 2c shows the PI of three island-diaphragm structures during evolution, which progressively increased throughout the evolution process. The final structure has a PI of 246, which means that the maximum stress of the optimum structure was 246% more than that of the flat diaphragm with the same deflection.

### Optimization result

**Fig. 3:**
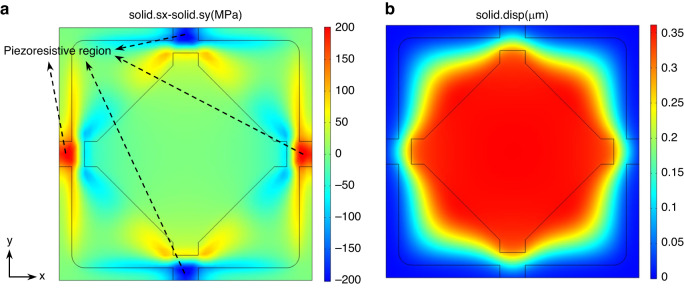
Optimization result. FEM simulation of (**a**) the transverse-to-longitudinal stress difference and (**b**) the deflection of the optimal island-diaphragm

Figure 3a shows the FEM simulation of the transverse-to-longitudinal stress difference of the optimal island-diaphragm, which has been modified in detail to facilitate fabrication compared to the optimization result. The figure shows that the central island concentrated the majority of the stress in the narrow piezoresistive region above the edge gaps, thereby preventing the stress from dissipating and maximizing the stress in the corresponding region to approximately 200 MPa. Such stress was sufficient to produce a high sensitivity of the sensor. Figure 3b shows the FEM simulation of the deflection of the optimal island-diaphragm. Due to the presence of the central island, the deformation of the central diaphragm was considerably constrained, while most of the deformation in the *x* and *y* directions was locally concentrated to the edge. In other directions, however, the space was relatively large, allowing for greater deformation and preventing an excessive increase in diaphragm stiffness. In such a design, the maximum deflection of the diaphragm was only 0.36 μm, approximately 1% of the diaphragm thickness, minimizing the nonlinearity generated by the deflection of the diaphragm.

**Fig. 4:**
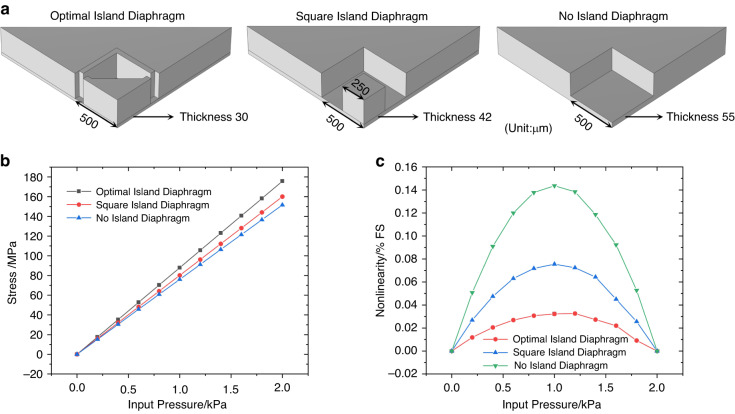
Comparison of three different island-diaphragm structures. **a** The stress on the diaphragm and (**b**) the corresponding nonlinearity (endpoint) as the function of input pressure

Figure 4 shows a comparison of three different island-diaphragm structures: the optimal island diaphragm, the square island diaphragm, and the no-island diaphragm. To facilitate the comparison of nonlinearity, the thicknesses of the flat portions of three diaphragms were adjusted to ensure that their surface stresses were similar. As shown, the stress on the optimal island diaphragm was 10% larger than the stress on the square island diaphragm and 16% larger than the stress on the no-island diaphragm for the specified dimensions. Nevertheless, the optimal island diaphragm had the lowest nonlinearity of stress, which was 43% of the nonlinearity of the square island diaphragm and 23% of the no-island diaphragm. It is clear that the designed optimal island diaphragm has a significant impact in increasing stress and decreasing nonlinearity.

### Fabrication and Characterization

**Fig. 5:**
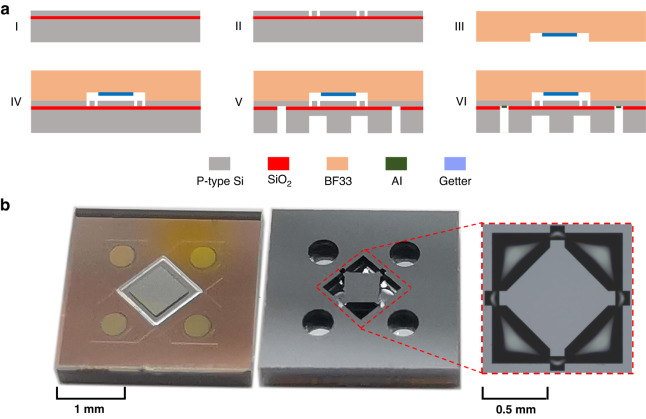
Fabrication of the fabricated PPS with optimal island-diaphragm. **a** Fabrication processes include key steps of: (I) cleaning the SOI wafer, (II) etching the device layer to form the Wheatstone bridge, (III) etching glass wafer and evaporating the getter to form the vacuum cavity, (IV) anodic bonding, (V) etching the handle layer to form the island-diaphragm and TSV and (VI) evaporating Al electrodes. **b** The front and back views of the fabricated PPS chip with details of the island-diaphragm

Next, the PPS with an optimal island-diaphragm was fabricated using a standard MEMS procedure. Figure 5a shows the fabrication processes. First, the SOI was thoroughly cleaned using concentrated sulfuric acid and hydrogen peroxide to remove impurities (Fig. 5a(I)). Then, the device layer of SOI was etched by deep reactive ion etching (DRIE) to form the Wheatstone bridge (Fig. 5a(II)). After, the BF33 glass wafer was etched with HF, while the getter was evaporated inside the cavity to serve as a vacuum reference (Fig. 5a(III)). The glass wafer and SOI were subsequently bonded using anodic bonding (Fig. 5a(IV)), and island-diaphragm and through silicon vias (TSV) were fabricated using DRIE on the substrate layer of SOI (Fig. 5a(V)). Finally, Al electrodes were evaporated over the TSV to extract the signal from the Wheatstone bridge on the device layer (Fig. 5a(VI)). Figure 5b shows the fabricated PPS chip with dimensions of 3 mm×3 mm×0.6 mm, and the island diaphragm is highlighted in the detailed diagram.

**Fig. 6:**
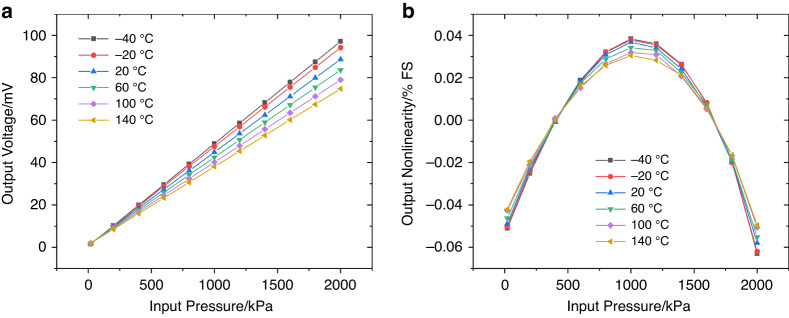
Characterization of the fabricated PPS. **a** The output voltage and (**b**) nonlinearity (least squares) of the sensor as functions of temperatures and pressures

The sensor was characterized under conditions of 20 to 2000 kPa external pressure, −40 to 140 °C ambient temperature, and 5 V DC excitation. Figure 6 shows the output voltage and nonlinearity (least squares) of the sensor as functions of temperature and pressure. The sensor exhibited a great sensitivity of 8.8 mV V^ 1^ MPa^-1^ and an extraordinary least squares nonlinearity of 0.058% FS at 20 °C. Even at temperatures ranging from −40 to 140 °C, the sensitivity could also be maintained above 7.4 mV V^−1^ MPa^-1^, while the nonlinearity was kept below 0.063% FS, validating the functionality of the proposed island-diaphragm structure.

Table 1 shows a comparison of performance results between the fabricated sensor and other sensors with different diaphragm structures, which demonstrates the outstanding sensitivity and nonlinearity of the fabricated sensor.

**Table 1:** Comparison of sensor performance

Sensor	This article	Chuang Li^19^	Bian Tian^20^	Kulite^21^
Power supply	5 V	5 V	5 V	10 V
Pressure range	0–2 MPa	0–1 psi	0–5 kPa	0–3.5 MPa
Diaphragm structure	Optimal island	Rood beam	Cross beam	Square island
Full scale output	88 mV	150 mV	35 mV	100 mV
Nonlinearity	0.06%FS	0.25%FS	0.10%FS	0.10%FS

## Conclusion

In this paper, a novel semiautomatic evolutionary method was implemented for MEMS structural design. The optimization procedure comprises three steps: establish the mechanical model and divide the component to be optimized into numerous equal-sized unit elements; evaluate the attribute of each element to produce a matrix that represents the contribution of each element; and use this matrix as a guide to, optimize the structure until its maximum performance is reached. We applied the evolutionary method to optimize the structure of the PPS pressure-sensitive diaphragm without requiring input parameter values and prior knowledge. The result of three distinct initial structures yielding the same optimal structure demonstrates that this method is insensitive to the initial conditions and can thus successfully avoid the optimization solver converging to a local maximum. The simulation of the final structure and the experimental results of the manufactured device indicate that the optimal structure obtained by this method can enable increased stress on the diaphragm and significantly minimize its nonlinearity. These favorable improvements in performance demonstrating the value of our evolutionary design method.

## References

[1] Fiorillo AS, Critello CD, Pullano SA (2018). Theory, technology and applications of piezoresistive sensors: a review. Sens. Actuators A: Phys..

[2] Fan, S. C. *Sensing Technology and Application* (Beijing University of Aeronautics and Astronautics Press, 2010).

[3] Pramanik C, Saha H (2006). Low pressure piezoresistive sensors for medical electronics applications. Mater. Manuf. Process..

[4] Fleming W (2001). Overview of automotive sensors. IEEE Sens. J..

[5] Eaton W, Smith J (1997). Micromachined pressure sensors: review and recent developments. Smart Mater. Struct..

[6] Wu Z, Hjort K (2009). Microfluidic hydrodynamic cell separation: a review. Micro Nanosyst..

[7] Niu Z, Zhao YL, Tian B (2014). Design optimization of high pressure and high temperature piezoresistive pressure sensor for high sensitivity. Rev. Sci. Instrum..

[8] Vang, T. A. Research on a Novel Structure MEMS Piezoresistive Pressure Sensor, Doctoral dissertation, South China University of Technology, Guangzhou (2018).

[9] Ongkodjojo A, Tay FEH (2002). Global optimization and design for microelectromechanical systems devices based on simulated annealing. J. Micromech. Microeng..

[10] Wang C (2020). Design of freeform geometries in a MEMS accelerometer with a mechanical motion preamplifier based on a genetic algorithm. Microsyst. Nanoeng..

[11] Wang C (2022). Design of a large-range rotary microgripper with freeform geometries using a genetic algorithm. Microsyst. Nanoeng..

[12] Sui, F. et al. *Designing Weakly Coupled Mems Resonators with Machine Learning-Based Method* 454–457 (IEEE MEMS 2022).

[13] Li Q (2021). A novel high-speed and high-accuracy mathematical modeling method of complex MEMS resonator structures based on the multilayer perceptron neural network. Micromachines.

[14] Guo R (2022). Deep learning for non-parameterized MEMS structural design. Microsyst. Nanoeng..

[15] Smith CS (1954). Piezoresistance effect in germanium and silicon. Phys. Rev..

[16] Bao, M. *Analysis and Design Principles of Mems Devices* (Elsevier, 2005).

[17] Clark SK, Wise KD (1979). Pressure sensitivity in anisotropically etched thin-diaphragm pressure sensors. IEEE Trans. Electron Devices.

[18] Sandmaier H, Kuhl K (1993). A square-diaphragm piezoresistive pressure sensor with a rectangular central boss for low-pressure ranges. IEEE Trans. Electron devices.

[19] Li C (2020). Characterization and analysis of a novel structural SOI piezoresistive pressure sensor with high sensitivity and linearity. Microsyst. Technol..

[20] Tian B (2012). The design and analysis of beam-membrane structure sensors for micro-pressure measurement. Rev. Sci. Instrum..

[21] Kulite Semiconductor Products, Inc. XTL-190 (M) SERIES Datasheet, https://kulite.com//assets/media/2021/02/HKM-198-375.pdf.

